# Accuracy of the Berger-Exner test for detecting third-order selection bias in randomised controlled trials: a simulation-based investigation

**DOI:** 10.1186/1471-2288-14-114

**Published:** 2014-10-06

**Authors:** Steffen Mickenautsch, Bo Fu, Sheila Gudehithlu, Vance W Berger

**Affiliations:** SYSTEM Initiative/Department of Community Dentistry, Faculty of Health Sciences, University of the Witwatersrand, 7 York Rd., Parktown, Johannesburg, 2193 South Africa; Department of Biostatistics, GSPH, University of Pittsburgh, Pittsburgh, PA USA; National Cancer Institute, National Institute of Health, Rockville, MD USA; Department of Statistics, University of Illinois at Urbana-Champaign, Champaign, IL USA

**Keywords:** Randomised trials, Selection bias, Berger-Exner test, Sensitivity, Specificity, ROC curve

## Abstract

**Background:**

Randomised controlled trials (RCT) are highly influential upon medical decisions. Thus RCTs must not distort the truth. One threat to internal trial validity is the correct prediction of future allocations (selection bias). The Berger-Exner test detects such bias but has not been widely utilized in practice. One reason for this non-utilisation may be a lack of information regarding its test accuracy. The objective of this study is to assess the accuracy of the Berger-Exner test on the basis of relevant simulations for RCTs with dichotomous outcomes.

**Methods:**

Simulated RCTs with various parameter settings were generated, using R software, and subjected to bias-free and selection bias scenarios. The effect size inflation due to bias was quantified. The test was applied in both scenarios and the pooled sensitivity and specificity, with 95% confidence intervals for alpha levels of 1%, 5%, and 20%, were computed. Summary ROC curves were generated and the relationships of parameters with test accuracy were explored.

**Results:**

An effect size inflation of 71% - 99% was established. Test sensitivity was 1.00 (95% CI: 0.99 – 1.00) for alpha level 1%, 5%, and 20%; test specificity was 0.94 (95% CI: 0.93 – 0.96); 0.82 (95% CI: 0.80 – 0.84), and 0.56 (95% CI: 0.54 – 0.58) for alpha 1%, 5%, and 20%, respectively. Test accuracy was best with the maximal procedure used with a maximum tolerated imbalance (MTI) = 2 as the randomisation method at alpha 1%.

**Conclusions:**

The results of this simulation study suggest that the Berger-Exner test is generally accurate for identifying third-order selection bias.

**Electronic supplementary material:**

The online version of this article (doi:10.1186/1471-2288-14-114) contains supplementary material, which is available to authorized users.

## Background

Bias has been defined as systematic error or deviation from the truth in results or inferences [1]. Selection bias is one form of systematic error that interferes with the internal validity of clinical trials [2] and may lead to favouring one treatment group over another [3]. This bias is based on knowledge about patient characteristics that are known to be conducive to successful trial outcomes together with foreknowledge regarding the allocation of such patients in a specific sequence of interventions (i.e., test and control interventions). Accordingly, such patients may consequently be scheduled to receive the particular intervention, thus creating a more favourable outcome than that of any other type of intervention (i.e., the control intervention). Theoretically, generation of a random allocation sequence in randomised control trials (RCT) balances all known and unknown patient characteristics (and risk factors) between groups and may thus ensure an equal chance for any patient, with any characteristic, to receive either type of intervention. However, such random allocation may be subverted: either when trial subjects are selected before generation of the random allocation sequence; the random allocation sequence is directly observed, or when the random allocation sequence is correctly predicted before subject allocation. Such types of subversion have been labelled as the introduction of first-, second- and third-order selection bias, respectively [2].

“Third-order residual” selection bias is present when, despite the application of what may generally be considered to be an adequate allocation concealment process, the result of this process was unsuccessful and subversion has occurred; i.e., through correct prediction of the random sequence allocation [2]. In such a situation opaque envelopes and/or central randomisation may be used to prevent direct observation of the random sequence, but future allocations can still be predicted, even if not observed directly. This may most easily be understood in terms of an unmasked trial that uses permuted blocks of size two, because here, even if the allocation sequence is never directly observed, it is still easy to determine every second allocation, since it must differ from the one just before it.

In this context, correct prediction of the random allocation sequence is a prerequisite for selection bias. Without prediction of future allocations, no third-order residual selection bias is possible but although a situation in which future allocations are predicted can be imagined, these predictions are not acted upon. The focus of this study is the situation in which this prediction *is* acted upon. In this case, the investigators may select healthier patients, or those patients more likely to respond well, when their treatment is due up, and sicker patients when the control is due up. The fact that those parties with the greatest vested interest in the outcome of the trial are the very ones conducting the trial makes it clear that there is an incentive to use whatever discretion may be available to ensure that the desired outcome is obtained.

It should not be surprising therefore, that overestimation of a treatment effect through bias has been observed to be more common than underestimation [4]. Moreover, Berger (2005) documents no fewer than 30 actual trials exhibiting characteristics of this type of selection bias [2]. Selected empirical evidence regarding the effect of selection bias shows an overestimation of treatment effects caused by lack of adequate random sequence generation and allocation, as well as by a lack of adequate allocation concealment, of 30% [5] to 51% [6], and of 13.3% [7] to 54% [8], respectively. Clearly, then, selection bias is a major problem, and needs to be controlled. Such control may take the form of prevention [9], detection [10] or correction [11]. All three approaches are important [2].

Prior to the development of the Berger-Exner test in 1999 [10], only basic methods were available for detecting selection bias. These would include the baseline comparisons that are standard in clinical trial reports. The Berger-Exner test is the first analysis that aims to directly detect selection bias by studying the mechanism through which it occurs (or does not occur, as the case may be). However, the result of a simple literature search in PubMed and GoogleScholar (30.09.2013), using “Berger-Exner test” as search term, suggests that since its development the test has been adopted as part of trial methodology in only six RCTs [12–17] and one RCT protocol [18], so far. These trials and the protocol were published during an eight-year period from 2005 – 2013 and cover topics in the fields of psychology [12] and internal medicine [14, 15, 18] and investigations related to drug addiction [13] and to HIV/AIDS [16, 17]. The trials were implemented by trialists, mostly based in the USA [12–14, 16, 17], but also in South Africa, the UK and Jamaica [14]; Italy, Switzerland and Turkey [15]; India [16] and Australia [17]. The need for further in-depth investigations notwithstanding, the results of a subsequent literature search in PubMed (01.10.2013), with names of the trialists as search terms, appear to suggest that these trialists did not use the Berger-Exner test in previous or subsequent RCTs.

Reasons for the apparent low utilization of the Berger-Exner test in RCTs may be ascribed to a lack of information regarding its test accuracy. Against this background, the aim of this study was to assess test accuracy on the basis of relevant simulations for RCTs with dichotomous outcomes.

## Methods

In order to investigate the accuracy of the Berger-Exner test for detecting third-order selection bias in RCTs, the numbers of true positive (TP), true negative (TN), false positive (FP), and false negative (FN) results were observed from RCT simulations. A ‘true positive’ test result (TP) was observed when the test indicated a positive result in the presence of bias and a ‘true negative’ test result (TN) was observed when the test indicated a negative result in the absence of bias. Accordingly, a ‘false positive’ test results (FP) was observed when the test indicated a positive result in the absence of bias and a ‘false negative’ test result (FN) was observed when the test indicated a negative result in the presence of bias. These numbers were converted into two rates: the test sensitivity and test specificity. The test sensitivity is defined as the proportion of cases with third-order selection bias in relation to the total number of cases that show positive test results, and the test specificity is defined as the proportion of cases without third-order selection bias in relation to the total number of cases that show negative test results [19]. For high accuracy it is ideal that sensitivity and specificity have somewhat reasonably high percentages, i.e., > 80%. In order to obtain a summarized predictive value of the two rates, the Diagnostic Odds Ratio (DOR) was computed. The DOR combines sensitivity and specificity into one single predictive summary measure and is defined as: DOR = (TP × TN)/(FP × FN) [19]. The DOR may range from zero to infinity, has no pre-defined cut-off threshold and is utilized for comparing the predictive evidence strength of different diagnostic parameter settings. A DOR value of, or close to 1.00 provides no predictive evidence and corresponds with the rising diagonal in Summary Receiver Operating Characteristic (SROC) graphs. The higher the DOR value (>1.00), the better the predictive accuracy [20].

Seven different randomisation procedures for each of three “trait frequencies” within each of the three sample sizes (N) were included. The trait frequency (TF) was defined as the percentage of subjects with a characteristic/trait ‘X’. Such trait was assumed to function as a confounding factor that would cause intervention success (Y = 1), regardless of the type of intervention group.

### Trial simulation and parameters

For the purpose of this study an RCT simulation was obtained by assuming the comparison of two interventions (Intervention A and B) with dichotomous outcomes (Intervention failure: Y = 0; Intervention success: Y = 1). A simulated RCT consisted of three components: (i) a sequence of subject ID (accession) numbers; (ii) a sequence of the Reverse Propensity Score (RPS) [11] per subject ID with regard to the propensity of the subject to be allocated to Group A; and (iii) a sequence of dichotomous outcomes per subject ID (Y = 1 or 0). The RPS reflects the probability of allocation of a patient to group A [2]. For example, with block size 4 and an [ABAB] block, the sequence of RPS values would be 2/4, 1/3, ½, 0/1, respectively, reflecting the ratio of the number of remaining A allocations within the block to overall remaining allocations within the block.

The parameters Trait Frequency (TF), subject number (N) and type of randomisation method (RM) were introduced into the simulation (Table 1). The seven randomisation methods were: fixed block randomisation with block size 4, 6, or 8; block randomisation with randomly varying block size 4, 6, 8 with equal probability (1/3) and the maximal procedure [9] with a maximum tolerated imbalance (MTI) of 2, 3, and 4. The Trait frequency (TF) was set to be 10%, 20% or 50% of the total number of subjects (N), which in turn was set to be 120, 240 or 480 subjects.

**Table 1:** **Generated parameter sets for both scenarios**

PSN	RM	N	TF/n	PSN	RM	N	TF/n
01	Fixed/BS = 4	120	10/12	33	Varying	240	50/120
02			20/24	34		480	10/48
03			50/60	35			20/96
04		240	10/24	36			50/240
05			20/48	37	Maximal procedure/MTI = 2	120	10/12
06			50/120	38			20/24
07		480	10/48	39			50/60
08			20/96	40		240	10/24
09			50/240	41			20/48
10	Fixed/BS = 6	120	10/12	42			50/120
11			20/24	43		480	10/48
12			50/60	44			20/96
13		240	10/24	45			50/240
14			20/48	46	Maximal procedure/MTI = 3	120	10/12
15			50/120	47			20/24
16		480	10/48	48			50/60
17			20/96	49		240	10/24
18			50/240	50			20/48
19	Fixed/BS = 8	120	10/12	51			50/120
20			20/24	52		480	10/48
21			50/60	53			20/96
22		240	10/24	54			50/240
23			20/48	55	Maximal procedure/MTI = 4	120	10/12
24			50/120	56			20/24
25		480	10/48	57			50/60
26			20/96	58		240	10/24
27			50/240	59			20/48
28	Varying	120	10/12	60			50/120
29			20/24	61		480	10/48
30			50/60	62			20/96
31		240	10/24	63			50/240
32			20/48				

PSN = Parameter set number; RM = Randomisation method; TF = Trait frequency; N = Subject number; n = Number of subjects per TF; BS = Block size; MTI = Maximum tolerated imbalance; Fixed = Fixed block randomisation.

Varying = Block randomisation with randomly varying block size 4, 6, 8 with equal probability (1/3).

### Study scenarios

As both interventions were assumed to have no effect, thus would not lead to ‘success’ on its own, the distribution of subjects with trait ‘X’ (Y = 1) among groups A and B served as an indicator for the presence/absence of selection bias. When subject allocation strictly followed a true random sequence, all subjects with trait ‘X’ were evenly distributed between groups A and B and neither intervention group was superior to the other (= Scenario 1: No selection bias). Subversion of the random allocation by correct prediction of the random sequence through use of the RPS and knowledge about which of the subjects carry trait ‘X’ allowed allocation of these subjects in favour of intervention group A (= Scenario 2: third-order selection bias). In this scenario, intervention A was superior to intervention B solely by virtue of an uneven distribution of subjects with trait ‘X’ (Y = 1), being equal to the specified TF.

Scenario 1 was simulated, using R statistical software, by assigning the first (N*TF)/100% of participants to Y = 1, and all others to Y = 0. Scenario 2 was simulated, using R statistical software, by assigning Y = 1 in accordance with the highest RPS, favouring intervention A above B as per TF. For each parameter set, 25 individual random sequences (‘runs’) were generated.

In order to illustrate the ‘effect size inflation due to third-order selection bias’ at the various parameter settings of TF and N in the RCT simulations, fixed effect meta-analysis was conducted of all simulated RCTs per TF/N setting, using RevMan 4.2.10 statistic software. Pooled Odds ratios (OR) with 95% Confidence Intervals (CI) were computed from dichotomous datasets consisting per intervention group of (i) the number of subjects with intervention failure, Y = 0 and (ii) the total number of subjects per TF/N setting. A Ratio of Odds Ratios (ROR) was calculated, being the Odds ratio (OR) of all datasets of Scenario 2 divided by the OR of Scenario 1. In absence of bias (Scenario 1) the OR is 1.00, indicating no difference in intervention failures between test- and control group. An OR below 1.00 indicates less intervention failures in the test group and an OR above 1.00 indicates less intervention failures in the control group. In Scenario, 2 bias favours the test group over that of the control group, i.e.: having less numbers of failed intervention (n), thus resulting in a lower OR. Because the OR of Scenario 1 is lager than the OR of Scenario 2, the calculated ROR is necessarily lower than 1.00. In line with convention [21], an ROR less than 1.0 indicates an overestimation in the effect size of the former group of datasets in comparison to the effect size of the referent group of datasets. From the thus established ROR, an effect size overestimation in percent, [1 – ROR] × 100%, was calculated. All OR were computed using RevMan 4.2.10 statistic software from the subject number (N) and numbers of failed intervention (n) per intervention group.

As the actual effect of both interventions was of no interest within this context, it was set at zero (Y = 0). Hence, when compared with each other, neither intervention would yield any result superior to that of the other (= Odds ratio 1.00). Such setting allowed investigation of the bias effect alone.

From the various parameters (Randomisation method, TF and N) a total of 63 different parameter sets for the simulated RCTs were generated and are presented in Table 1. All RCT simulations were conducted in four steps, using R statistical software, based on the generated variables: ID, BLOCK, TRT, RPS, Y

- Step 1: Generation of subject identification (ID) i = 1:N;
- Step 2: Generation of randomisation-blocks/MTI (BLOCK) and randomisation according to different RM);
- Step 3: Generation of 2-arm treatment (TRT) in each block. For fixed and varying block randomisation, two treatments have equal probability to be assigned to either group, and within each block, the number of each treatment should be the same, Thus random numbers were generated from a standard uniform distribution for a simulated RCT with block size m_i_, i = 1,…,N, m/2 of which those above the median were assigned as intervention A (TRT = 1), and all other m/2 were assigned as intervention B (TRT = 0). For the maximal procedure (MP), the generation of treatment sequence was based on the MTI [9]; i.e., the qualified random sequence should satisfy I(D) < = MTI and I_N_(D) = 0, where I_k_(D) = |S_k,A_(D)-S_k,B_(D)|, and S_k,A_(D) = sum(X_i_(D)), I = 1,…,k, and S_k,B_(D) = k- S_k,A_(D), where X_i_(D) = 1, if sequence D assigns the i^th^ patient to treatment group A, X_i_(D) = 0, if sequence D assigns the i^th^ patient to treatment group B.
- Step 4: Calculation of the RPS from the generated block and treatment, using code “rps.gen” in R (Additional file 1: Appendix file 1) on the basis of the block information (BLOCK) and the treatment information (TRT).

### Bias testing

The Berger-Exner test has been applied for bias testing. The test consisted of linear regression analysis, separately per treatment group, including the RPS as independent, and the Y-values as dependent variables [2]. Regression analysis was conducted separately per intervention group and the resulting p-values were recorded. In order to investigate the influence of various alpha levels on the test accuracy, alpha was set at 1%, 5% and 20%.

A true negative (TN) result was established when both p-values for intervention group A and B were above 0.01; 0.05 or 0.20 for alpha 1%, 5% and 20% (two-sided), respectively. A true positive (TP) result was established when at least one of the p-values of either intervention group was below 0.01; 0.05 or 0.20 for alpha 1%, 5% and 20%, respectively. The number of false positive (FP) results was calculated by subtracting the total number of TN from the total number of runs per parameter set; i.e., FP = 25 – TN. The number of false negative (FN) results was calculated by subtracting the total number of TP from the total number of runs per parameter set; i.e., FN = 25 – TP.

### Data analysis and summary measures

From the 63 separate parameter sets, the established total numbers of true negative/false positive (TN/FP) and true positive/false negative (TP/FN) results, per set alpha level, were entered into Meta-DiSc Version 1.4 statistical software [22] and the pooled specificity and sensitivity with 95% Confidence Intervals (CI) for each alpha level were computed. In addition, symmetrical Summary Receiver Operating Characteristic (SROC) curves per alpha level were generated from this data. The SROC curve shows the relationship of the sensitivity and the complement of the specificity for all the individual test results; i.e., the fractional relationship between TP (TP/(TP + FN)) and FP (FP/(FP + TN)).

The influence of parameters N, TF and RM was investigated by computing the Diagnostic Odds Ratio (DOR with 95% CI) from the relevant TN/FP and TP/FN data per parameter setting of each study parameter per alpha level (Table 1), using Meta-DiSc Version 1.4 statistical software.

All data pooling, using Meta-Disc 1.4 software, was based on the standard Der Simonian Laird random-effects model [22]. The DerSimonian Laird method produces a random-effects meta-analysis that incorporates an assumption that different studies are estimating different, yet related, effects. The model may not be optimal but remains valid even when the random effects are not normally distributed. In addition, the model allows the treatment effects to differ across runs, with an underlying true effect, and a between-runs variance, by using a non-iterative model to estimate the treatment effect variance.

## Results

For the purpose of this study the true effect size of the simulated RCT results was set at OR 1.00. Third order selection bias effected an inflation of the true effect size to a range between ROR 0.01 – 0.29, thus reflecting an overestimation ranging from 71% - 99%. Table 2 presents the Odds ratios (OR) that were computed from the pooled number of subjects with failed intervention (n) and the total subject numbers (N), as well as the calculated ROR and percent overestimation (OE%) per parameter setting for Scenario 1 and 2.

**Table 2:** **Inflation of effect size due to 3** ^**rd**^ **order selection bias**

Parameter setting	Scenario 1*						Scenario 2**						ROR/OE%
	Group A		Group B		OR	95% CI	Group A		Group B		OR	95% CI	
	n	N	n	N			n	N	n	N			
TF = 10% - N = 120	19950	21000	19950	21000	1.00	0.92 – 1.09	18912	21000	20988	21000	0.04	0.03 – 0.05	0.04/96%
TF = 10% - N = 240	31500	42000	31500	42000	1.00	0.94 – 1.06	37824	42000	41976	42000	0.02	0.02 – 0.03	0.02/98%
TF = 10% - N = 480	63000	84000	63000	84000	1.00	0.96 – 1.04	75624	84000	83976	84000	0.01	0.01 – 0.01	0.01/99%
TF = 20% - N = 120	18900	21000	18900	21000	1.00	0.94 – 1.07	17117	21000	20681	21000	0.09	0.08 – 0.10	0.09/91%
TF = 20% - N = 240	37800	42000	37800	42000	1.00	0.96 – 1.07	34242	42000	41343	42000	0.08	0.07 – 0.09	0.08/92%
TF = 20% - N = 480	75600	84000	75600	84000	1.00	0.97 – 1.03	68493	84000	82717	84000	0.08	0.07 – 0.08	0.08/92%
TF = 50% - N = 120	15750	21000	15750	21000	1.00	0.96 – 1.05	13417	21000	18093	21000	0.29	0.27 – 0.30	0.29/71%
TF = 50% - N = 240	39900	42000	39900	42000	1.00	0.97 – 1.03	26858	42000	36150	42000	0.29	0.28 – 0.30	0.29/71%
TF = 50% - N = 480	79800	84000	79800	84000	1.00	0.98 – 1.02	53812	84000	72178	84000	0.29	0.28 – 0.30	0.29/71%

TF = Trait frequency; N = Subject number; OR = Pooled Odds ratio; CI = Confidence interval; n = Pooled number of subjects with failed intervention (Y = 0); N = Pooled total umber of subjects;

ROR = Ratio of Odds ratios; OE% = Percent overestimation/inflation of true effect size.

*No bias; **3^rd^ order selection bias.

A total of 3150 runs were conducted, 1575 for each scenario. From these, testing in scenario 1 yielded 1488; 1294 and 883 true negative (TN) results for alphas 1%; 5% and 20%, respectively. Testing in scenario 2 yielded 1574; 1574 and 1575 true positive (TP) results for alphas 1%; 5% and 20%, respectively (Additional file 2: Appendix file 2).

### Test sensitivity and specificity

**Figure 1:**
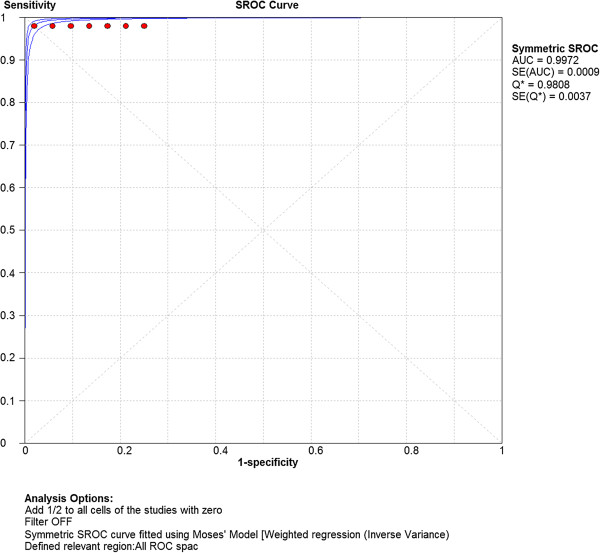
**Summary Receiver Operating Characteristic (SROC) curve concerning test accuracy at alpha level 1%.** AUC = Area under curve; SE = Standard error.

**Figure 2:**
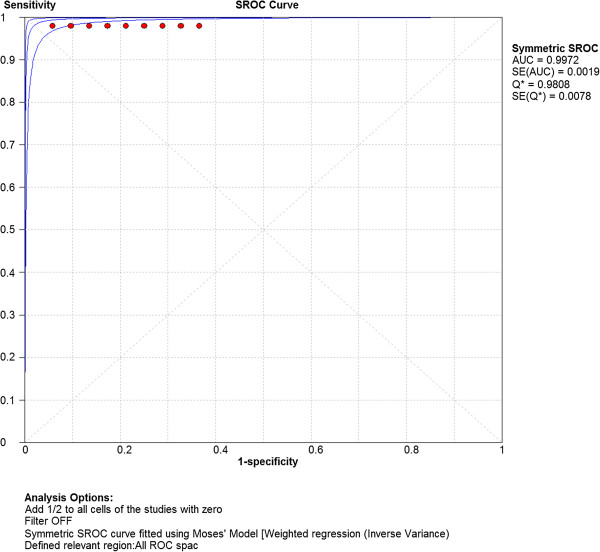
**Summary Receiver Operating Characteristic (SROC) curve concerning test accuracy at alpha level 5%.** AUC = Area under curve; SE = Standard error.

**Figure 3:**
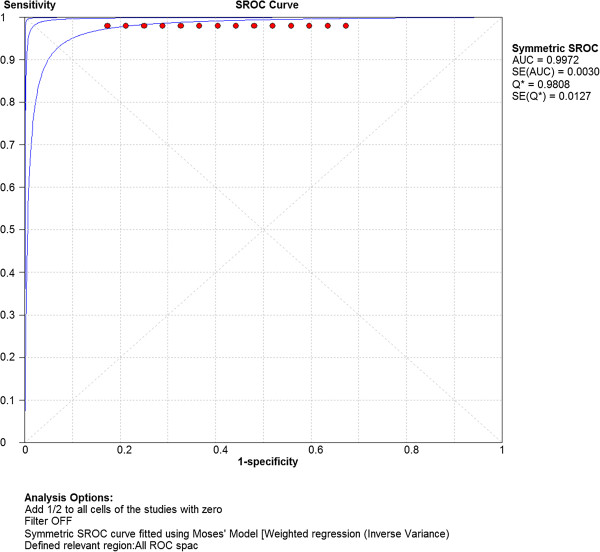
**Summary Receiver Operating Characteristic (SROC) curve concerning test accuracy at alpha level 20%.** AUC = Area under curve; SE = Standard error.

Computation of the pooled test sensitivity and specificity was based on the TP/FN and TN/FP data from the 63 separate parameter sets (Table 1, Additional file 2: Appendix file 2), per alpha level. The pooled sensitivity was 1.00 (95% CI: 0.99 – 1.00) for alpha level 1%, 5% and 20%. The pooled test specificity was 0.94 (95% CI: 0.93 – 0.96) for alpha level 1% and 0.82 (95% CI: 0.80 – 0.84) and 0.56 (95% CI: 0.54 – 0.58) for alpha 5% and 20%, respectively. The generated SROC curves (Figures 1, 2, 3) indicated highest overall test accuracy when alpha was set at 1%.

### Association of trial parameters with test accuracy

Diagnostic Odds Ratios (DOR) were computed per alpha level based on the TP/FN and TN/FP data from 21 relevant parameter sets of parameter settings for the number of subjects (N) and trait frequency (TF), as well as from 9 relevant parameter sets of settings for randomisation method (RM) (Table 1, Additional file 2: Appendix file 2). The results are presented in Table 3.

**Table 3:** **Pooled diagnostic Odds ratios (DOR) per study parameter**

Study parameter	Parameter setting	Alpha 1%		Alpha 5%		Alpha 20%	
		DOR*	95% CI*	DOR*	95% CI*	DOR*	95% CI*
N	120	835	396 - 1763	212	110 - 406	62	33 - 117
	240	698	334 - 1455	242	124 - 474	71	37 - 135
	480	668	325 - 1374	234	122 - 451	65	34 - 122
TF	10%	948	443 - 2027	238	124 - 458	64	34 - 120
	20%	785	378 - 1626	251	130 - 484	69	37 - 131
	50%	547	272 - 1101	204	107 - 391	64	34 - 120
RM	Fixed/BS = 4	995	311 - 3186	500	176 - 1420	113	43 - 299
	Fixed/BS = 6	666	225 - 1976	233	86 - 630	86	32 - 227
	Fixed/BS = 8	566	196 - 1638	185	69 - 498	63	24 - 166
	Varying	723	242 - 2166	182	69 - 494	52	20 - 136
	MP/MTI = 2	1456	432 - 4906	395	108 - 805	72	27 - 189
	MP/MTI = 3	712	239 - 2126	224	83 - 604	53	20 - 140
	MP/MTI = 4	474	160 - 1404	144	54 - 382	43	16 – 114

CI = Confidence Intervals; Fixed = Fixed block randomisation; BS = Block size; MTI = Maximum tolerated imbalance; Fixed = Fixed block randomisation; Varying = Block randomisation with randomly varying block size 4, 6, 8 with equal probability (1/3); MP = Maximal procedure; MTI = Maximum tolerated imbalance; N = Subject number; TF = Trait frequency;

RM = Randomisation method.

*Decimals rounded.

Higher accuracy was observed for all parameters when alpha was set at 1%. In comparison to all other randomisation methods, the results further suggest highest test accuracy with maximal procedure [9] with MTI = 2 as randomisation method (DOR 1456; 95% CI: 432 – 4906) at alpha 1%. For all alpha levels, test accuracy appeared to be associated with lower block size (BS) when fixed randomisation was used, but not with subject number (N) or trait frequency (TF).

## Discussion

The aim of this study was to assess the accuracy of the Berger-Exner test in identifying ‘third-order residual selection bias’ on the basis of relevant simulations for RCTs with dichotomous outcomes. As the accuracy was not tested under real RCT conditions, the outcomes of this study may have been limited by several methodological factors.

### Limitations of the current study method

In this simulation study only one single confounding factor, ‘trait X’, was utilized. This can be regarded as an oversimplification because many unknown confounding factors that can influence trial outcomes may indeed exist. These factors may interact in an enhancing or suppressive manner and their compound influence on the study results may lead to over- or underestimation of any intervention types studied. Alternatively, such factors may also have no impact at all. The ‘trait frequency’ that was simulated for the study samples may also not always be specific, but may have varying value.

The assumption of equivalence between both interventions was set as an absolute (= Odds ratio 1.00). However, even if interventions with almost the same treatment effects were compared, such effects would seldom be of absolute equivalence, even if no statistically significant differences were observed. Furthermore, the relationship of sample size to observed effect estimates in real world randomised control trials may affect test accuracy. For these reasons the perfect settings of the chosen simulation method may have contributed to artificially increased test accuracy.

Notwithstanding such limitations, the chosen trial simulation could establish the rationale of test accuracy of the Berger-Exner test by providing first insights as to which aspects, present in randomised control trials, may affect its utility.

### Discussion of results

As RCTs are highly influential in medical decisions, they must not distort the truth. One threat to internal trial validity is the correct prediction of future allocations (third-order selection bias). Against the background of high systematic error, due to third-order residual selection bias and quantified as effect-size overestimation ranging from 71 – 99% (Table 2), the results of this trial simulation show high sensitivity and specificity of the Berger-Exner test (Table 3) for RCTs with dichotomous outcomes. These results were affected by the chosen alpha level and block/MTI size, but not by trait-frequency level and sample size: While the test sensitivity values were the same for all alpha levels, the specificity values decreased with increasing alpha level. DOR values increased with lower alpha and lower block size/MTI.

How these results relate to the available evidence provided by real-world RCTs that have utilized the Berger-Exner test in the past remains unclear, as most of these investigated continuous outcomes: Depression scale and Mental health summary scores [12]; Addiction withdrawal severity scores [13]; percentage of days of disease [14]; mean absolute change in artery dilation [17]. For this reason it is difficult to apply the results of this simulation study to the reported settings and test results of these RCTs. The two RCTs that did include dichotomous outcomes in terms of disease and morbidity rates reported a negative Berger-Exner test at alpha level 5% and employed varying block randomisation [15] and fixed block randomisation with block size 8 [16]. The results of this simulation study suggest comparatively low test accuracy at these parameter settings with DOR 182 (95% CI: 69 – 494) and DOR 185 (95% 69 – 498), respectively (Table 3). Through the presented trial simulation, a mean percentage (SD = Standard deviation) of 79% (SD = 8%) true negative (TN) test results of all test results (see Additional file 2: Appendix file 2/PSN 19–27 and 28–36, respectively) was achieved for fixed block randomisation with block size 8 and varying block randomisation, each, at alpha 5%. However, the likelihood of such still reasonably high test accuracy for correctly identifying absence of third-order selection bias, may be lower in both RCTs [15, 16] (and thus, the reported absence of selection bias in these trials less reliable) as the test accuracy identified in this simulation study may be artificially increased, owing to the stated limitations of the chosen study method. However, due to the current lack of randomised control trials that have used the Berger-Exner test, more in-depth considerations as to the meaning of the reported test results cannot yet be made.

Notwithstanding the current limitations, a routine application of the Berger-Exner test in RCTs may have the advantage of providing a quantitative answer to the question as to whether the random allocation sequence concealment was effective or not. The ‘Risk of bias’ assessment tool, currently advocated by the Cochrane Collaboration does not provide such answer [23]. RCT reports cannot provide proof of absence of third-order selection bias during the trial simply by virtue of stating in detail the use of adequate concealment methods. Although, RCTs that reported adequate concealment were found to yield lower effect estimates than those with inadequate or unknown allocation concealment, such observations were limited as it was not possible to predict direction and magnitude of such effect-size changes [24]. Such uncertainty may partially be due to the possibility that some RCTs, which reported adequate concealment were compromised by correct prediction, without unmasking of the random allocation sequence during the trials.

An inclusion of the Berger-Exner test into routine RCT methodology, together with the reporting of underlying test data (dichotomous intervention outcome and RPS score per trial subject) and test result may thus aid in the correct distinction between RCTs with adequate or inadequate concealment. Such information may benefit possible statistical RPS-based bias correction [11, 25] and RCT evidence rating: Evidence rating systems, such as the GRADE system, have been developed for rating evidence quality and strength of recommendations derived from such evidence. The GRADE system in particular recommends quality downgrading of evidence from RCTs that lack adequate allocation concealment. However, it has been observed that empirical evidence in support of GRADE criteria is limited and that the GRADE system has been shown to give inconsistent results [26]. Additionally, the investigation of a quantitative approach to bias-risk assessment appears not to be the current focus of the Cochrane Bias Methods Group (BMG), so far [27]. For that reason, the Berger-Exner test may prove to be a useful tool in providing an empirical basis for RCT evidence rating.

### Recommendation for further research

Wide adoption of the Berger-Exner test into general RCT methodology of future studies could aid the verification of the current simulation results in practice. In addition, several aspects that require further investigation have emerged during the process of this study:

Even though the study simulation relied on binary values (Y = 0 / Y = 1) as the dependent variable, difficulties in using logistic instead of linear regression were observed. For scenario 2 (third-order selection bias), the situation may arise that for all subjects in one treatment group there may be only one value of outcome (Y = 0 or Y = 1). In such cases, logistical regression fails when Y is treated as the dependent variable. In contrast, linear regression treats the RPS value as the independent variable, which is why it was selected as the analytical method for use in this trial simulation.

Other aspects of this study also require further investigation. Some relate to the test accuracy in RCTs with continuous outcomes, as well as answers to questions as to why the observed test accuracy in trial simulations with dichotomous outcomes was higher with the maximal procedure than with other randomisation methods at alpha 1% and why the test accuracy was reduced at alpha 5% and 20%. Other questions still begging answers are related to reasons why test accuracy is reduced with higher block size/MTI, and to what the cause for false positive/false negative test results would be. Furthermore, the test accuracy established in this study requires verification at more real-world RCT simulation settings, such as varying effect estimates between competing interventions. Such future studies will show whether the ideal assumptions (i.e., absolute effect equivalence between interventions) on which the presented simulation method was based would, or would not have resulted in artificially increased test accuracy.

## Conclusion

The results of this simulation-based investigation suggest that the Berger-Exner test may successfully detect third-order selection bias at high accuracy under the condition that the maximal procedure with MTI = 2 as randomisation method is used with alpha set at 1%. However, some questions remain as to the empirical accuracy of this test and further research in this field is needed. Notwithstanding the limitations of this study, a routine adoption of the test into general RCT methodology is recommended as this may assist in the real-life corroboration of the simulation results and further investigation of test characteristics.

## Electronic supplementary material

Additional file 1: **Appendix 1 - R code “rps.gen”.** (DOC 19 KB)

Additional file 2: **Appendix 2 - Test results.** (DOC 138 KB)
