# Supplementary material for: Accuracy of the Berger-Exner test for detecting third-order selection bias in randomised controlled trials: a simulation-based investigation
Source: BMC Med Res Methodol. 2014 Oct 6;14:114. doi: 10.1186/1471-2288-14-114 (PMC4209086; doi:10.1186/1471-2288-14-114)
Supplement: Supplementary file 1 — Additional file 1: Appendix 1 - R code “rps.gen”. (DOC 19 KB) [file 12874_2013_1127_MOESM1_ESM.doc]

**Appendix file 1 -** R code “rps.gen”

**# Function to generate RPS, given treatment setting and block setting**

**RPS.gen <- function(trt, # Treatment setting, require binary here**

**block # Block setting, can be different block size**

**){**

**index <- unique(block)**

**rps <- NULL**

**for(i in 1:length(index)){**

**trt.sub <- trt[which(block==index[i])]**

**rps.sub <- sapply(1:length(trt.sub),**

**function(j){**

**ifelse(j==1, sum(trt.sub)/length(trt.sub),**

**sum(trt.sub[-(1:(j-1))])/length(trt.sub[-(1:(j-1))]))})**

**rps <- c(rps,rps.sub)**

**}**

**return(rps)**

}
