# Supplementary material for: Accuracy of the Berger-Exner test for detecting third-order selection bias in randomised controlled trials: a simulation-based investigation
Source: BMC Med Res Methodol. 2014 Oct 6;14:114. doi: 10.1186/1471-2288-14-114 (PMC4209086; doi:10.1186/1471-2288-14-114)
Supplement: Supplementary file 2 — Additional file 2: Appendix 2 - Test results. (DOC 138 KB) [file 12874_2013_1127_MOESM2_ESM.doc]

**Appendix file 2 -** Test results

|  | Alpha 1% | |  |  | Alpha 5% | |  |  | Alpha 20% | |  |  |
| --- | --- | --- | --- | --- | --- | --- | --- | --- | --- | --- | --- | --- |
|  | Scenario 1 | | Scenario 2 | | Scenario 1 | | Scenario 2 | | Scenario 1 | | Scenario 2 | |
| PSN | TN | FP | TP | FN | TN | FP | TP | FN | TN | FP | TP | FN |
| 1 | 25 | 0 | 25 | 0 | 24 | 1 | 25 | 0 | 17 | 8 | 25 | 0 |
| 2 | 23 | 2 | 25 | 0 | 21 | 4 | 25 | 0 | 14 | 11 | 25 | 0 |
| 3 | 23 | 2 | 25 | 0 | 23 | 2 | 25 | 0 | 18 | 7 | 25 | 0 |
| 4 | 25 | 0 | 25 | 0 | 24 | 1 | 25 | 0 | 17 | 8 | 25 | 0 |
| 5 | 23 | 2 | 25 | 0 | 20 | 5 | 25 | 0 | 17 | 8 | 25 | 0 |
| 6 | 23 | 2 | 25 | 0 | 22 | 3 | 25 | 0 | 16 | 9 | 25 | 0 |
| 7 | 25 | 0 | 25 | 0 | 24 | 1 | 25 | 0 | 20 | 5 | 25 | 0 |
| 8 | 25 | 0 | 25 | 0 | 24 | 1 | 25 | 0 | 18 | 7 | 25 | 0 |
| 9 | 25 | 0 | 25 | 0 | 24 | 1 | 25 | 0 | 19 | 6 | 25 | 0 |
| 10 | 25 | 0 | 25 | 0 | 19 | 6 | 25 | 0 | 13 | 12 | 25 | 0 |
| 11 | 25 | 0 | 25 | 0 | 23 | 2 | 25 | 0 | 20 | 5 | 25 | 0 |
| 12 | 23 | 2 | 25 | 0 | 22 | 3 | 25 | 0 | 19 | 6 | 25 | 0 |
| 13 | 23 | 2 | 25 | 0 | 22 | 3 | 25 | 0 | 17 | 8 | 25 | 0 |
| 14 | 24 | 1 | 25 | 0 | 20 | 5 | 25 | 0 | 15 | 10 | 25 | 0 |
| 15 | 23 | 2 | 25 | 0 | 18 | 7 | 25 | 0 | 13 | 12 | 25 | 0 |
| 16 | 22 | 3 | 25 | 0 | 21 | 4 | 25 | 0 | 11 | 14 | 25 | 0 |
| 17 | 24 | 1 | 25 | 0 | 22 | 3 | 25 | 0 | 17 | 8 | 25 | 0 |
| 18 | 23 | 2 | 25 | 0 | 19 | 6 | 25 | 0 | 16 | 9 | 25 | 0 |
| 19 | 25 | 0 | 25 | 0 | 20 | 5 | 25 | 0 | 10 | 15 | 25 | 0 |
| 20 | 22 | 3 | 25 | 0 | 19 | 6 | 25 | 0 | 16 | 9 | 25 | 0 |
| 21 | 23 | 2 | 25 | 0 | 18 | 7 | 25 | 0 | 10 | 15 | 25 | 0 |
| 22 | 24 | 1 | 25 | 0 | 19 | 6 | 25 | 0 | 13 | 12 | 25 | 0 |
| 23 | 24 | 1 | 25 | 0 | 24 | 1 | 25 | 0 | 21 | 4 | 25 | 0 |
| 24 | 21 | 4 | 25 | 0 | 18 | 7 | 25 | 0 | 13 | 12 | 25 | 0 |
| 25 | 23 | 2 | 25 | 0 | 18 | 7 | 25 | 0 | 13 | 12 | 25 | 0 |
| 26 | 24 | 1 | 25 | 0 | 20 | 5 | 25 | 0 | 9 | 16 | 25 | 0 |
| 27 | 23 | 2 | 25 | 0 | 21 | 4 | 25 | 0 | 18 | 7 | 25 | 0 |
| 28 | 25 | 0 | 25 | 0 | 22 | 3 | 25 | 0 | 13 | 12 | 25 | 0 |
| 29 | 24 | 1 | 25 | 0 | 18 | 7 | 25 | 0 | 12 | 13 | 25 | 0 |
| 30 | 22 | 3 | 25 | 0 | 18 | 7 | 25 | 0 | 16 | 9 | 25 | 0 |
| 31 | 24 | 1 | 25 | 0 | 18 | 7 | 25 | 0 | 10 | 15 | 25 | 0 |
| 32 | 24 | 1 | 25 | 0 | 20 | 5 | 25 | 0 | 11 | 14 | 25 | 0 |
| 33 | 22 | 3 | 25 | 0 | 19 | 6 | 25 | 0 | 14 | 11 | 25 | 0 |
| 34 | 23 | 2 | 25 | 0 | 18 | 7 | 25 | 0 | 10 | 15 | 25 | 0 |
| 35 | 24 | 1 | 25 | 0 | 21 | 4 | 25 | 0 | 13 | 12 | 25 | 0 |
| 36 | 25 | 0 | 25 | 0 | 23 | 2 | 25 | 0 | 14 | 11 | 25 | 0 |
| 37 | 25 | 0 | 25 | 0 | 20 | 5 | 25 | 0 | 16 | 9 | 25 | 0 |
| 38 | 25 | 0 | 25 | 0 | 23 | 2 | 25 | 0 | 17 | 8 | 25 | 0 |
| 39 | 23 | 2 | 25 | 0 | 19 | 6 | 25 | 0 | 12 | 13 | 25 | 0 |
| 40 | 25 | 0 | 25 | 0 | 22 | 3 | 25 | 0 | 16 | 9 | 25 | 0 |
| 41 | 25 | 0 | 25 | 0 | 23 | 2 | 25 | 0 | 16 | 9 | 25 | 0 |
| 42 | 24 | 1 | 25 | 0 | 22 | 3 | 25 | 0 | 13 | 12 | 25 | 0 |
| 43 | 24 | 1 | 25 | 0 | 22 | 3 | 25 | 0 | 16 | 9 | 25 | 0 |
| 44 | 25 | 0 | 25 | 0 | 22 | 3 | 25 | 0 | 13 | 12 | 25 | 0 |
| 45 | 25 | 0 | 25 | 0 | 21 | 4 | 25 | 0 | 13 | 12 | 25 | 0 |
| 46 | 25 | 0 | 25 | 0 | 22 | 3 | 25 | 0 | 12 | 13 | 25 | 0 |
| 47 | 24 | 1 | 25 | 0 | 21 | 4 | 25 | 0 | 14 | 11 | 25 | 0 |
| 48 | 23 | 2 | 25 | 0 | 18 | 7 | 25 | 0 | 11 | 14 | 25 | 0 |
| 49 | 24 | 1 | 25 | 0 | 22 | 3 | 25 | 0 | 17 | 8 | 25 | 0 |
| 50 | 23 | 2 | 25 | 0 | 21 | 4 | 25 | 0 | 13 | 12 | 25 | 0 |
| 51 | 25 | 0 | 25 | 0 | 22 | 3 | 25 | 0 | 11 | 14 | 25 | 0 |
| 52 | 22 | 3 | 25 | 0 | 20 | 5 | 25 | 0 | 13 | 12 | 25 | 0 |
| 53 | 24 | 1 | 25 | 0 | 21 | 4 | 25 | 0 | 16 | 9 | 25 | 0 |
| 54 | 23 | 2 | 25 | 0 | 18 | 7 | 25 | 0 | 8 | 17 | 25 | 0 |
| 55 | 25 | 0 | 25 | 0 | 19 | 6 | 25 | 0 | 11 | 14 | 25 | 0 |
| 56 | 25 | 0 | 25 | 0 | 18 | 7 | 25 | 0 | 8 | 17 | 25 | 0 |
| 57 | 20 | 5 | 25 | 0 | 17 | 8 | 25 | 0 | 8 | 17 | 25 | 0 |
| 58 | 25 | 0 | 25 | 0 | 21 | 4 | 25 | 0 | 13 | 12 | 25 | 0 |
| 59 | 22 | 3 | 25 | 0 | 21 | 4 | 25 | 0 | 12 | 13 | 25 | 0 |
| 60 | 23 | 2 | 25 | 0 | 19 | 6 | 25 | 0 | 15 | 10 | 25 | 0 |
| 61 | 19 | 6 | 25 | 0 | 16 | 9 | 25 | 0 | 13 | 12 | 25 | 0 |
| 62 | 20 | 5 | 25 | 0 | 16 | 9 | 25 | 0 | 9 | 16 | 25 | 0 |
| 63 | 24 | 1 | 25 | 0 | 20 | 5 | 25 | 0 | 14 | 11 | 25 | 0 |
|  |  |  |  |  |  |  |  |  |  |  |  |  |
| PSN | Parameter set number | | | |  |  |  |  |  |  |  |  |
| TN | True negative | | |  |  |  |  |  |  |  |  |  |
| FP | False positive | | |  |  |  |  |  |  |  |  |  |
| TP | True positive | | |  |  |  |  |  |  |  |  |  |
| FN | False negative | | |  |  |  |  |  |  |  |  |  |
